# Three-Dimensional Evaluation of TMJ Morphology in Individuals with Maxillary or Mandibular Impacted Canines: A CBCT-Based Retrospective Study

**DOI:** 10.3390/diagnostics16030496

**Published:** 2026-02-06

**Authors:** Fırat Oğuz, Samet Özden

**Affiliations:** Department of Orthodontics, Faculty of Dentistry, İnönü University, Malatya 44280, Türkiye; drsametozden@gmail.com

**Keywords:** TMJ, impacted maxillary canine, impacted mandibular canine, CBCT

## Abstract

**Background/Objectives:** This study aimed to evaluate temporomandibular joint (TMJ) morphology in individuals with impacted maxillary and mandibular canine teeth using cone beam computed tomography (CBCT) and to compare the findings with those of a control group without impacted canines. **Methods:** A total of 80 individuals were included in this retrospective study. Based on CBCT images, participants were divided into three groups: the impacted maxillary canine group (*n* = 30), impacted mandibular canine group (*n* = 20), and control group (*n* = 30). CBCT images were oriented in the 3D Slicer software according to the Frankfurt Horizontal plane and the midsagittal reference line. Condylar width, length, position, angular parameters, joint spaces, and condylar volume were measured. Appropriate parametric and non-parametric statistical tests were used for intergroup comparisons. **Results:** The control group exhibited significantly higher values of condylar width, coronal condylar position and angle, certain joint spaces, and condylar volume compared with both impacted maxillary and mandibular canine groups (*p* < 0.05). In particular, significant differences were observed for condylar width (*p* ≤ 0.002) (Control: 19.76 ± 2.09 mm, Maxillary: 17.92 ± 2.14 mm, Mandibular: 17.76 ± 1.64 mm), coronal condylar position (*p* < 0.001) (Control: 7.50 ± 1.34 mm, Maxillary: 6.02 ± 0.89 mm, Mandibular: 6.30 ± 0.83 mm), coronal condylar angle (*p* < 0.001) (Control: 25.09° ± 4.40, Maxillary: 28.80° ± 3.70, Mandibular: 33.37° ± 4.10), and condylar volume (*p* < 0.001) (Control: 1755.87 ± 357.32 mm^3^, Maxillary: 1337.18 ± 302.65 mm^3^, Mandibular: 1252.71 ± 369.24 mm^3^). No significant differences were found between the impacted maxillary and mandibular canine groups for most parameters (*p* > 0.05). Right–left side comparisons demonstrated that bilateral symmetry was largely preserved, except for condylar volume (*p* > 0.05). **Conclusions:** The presence of impacted canines may influence TMJ morphology, particularly at the level of condylar morphometry and joint spaces. Therefore, considering TMJ morphology in addition to local dental factors when evaluating impacted canines may provide a more comprehensive approach to orthodontic diagnosis and treatment planning.

## 1. Introduction

Maxillary canines represent the second most frequently impacted tooth group after third molars and are prone to ectopic eruption [1]. Their reported prevalence ranges between 1% and 3% [1,2]. In addition to causing esthetic and functional problems, impacted canines may induce root resorption of adjacent teeth, often necessitating orthodontic and surgical intervention [3]. In contrast, impacted mandibular canines are considerably rarer [4,5]. A systematic review published in 2017 reported an incidence of impacted mandibular canines ranging from 0.92% to 1.35% [4]. Whether located in the maxilla or mandible, impacted canines may compromise dental arch integrity, disrupt occlusal relationships, and alter functional force transmission, potentially leading to changes in the temporomandibular joint (TMJ) morphology.

The temporomandibular joint (TMJ) plays a critical role in the maintenance of craniomandibular functions. Its morphological structure and biomechanical balance form a dynamic system that directly influences essential functions such as mastication, speech, and swallowing. Conversely, these functional systems also interact bidirectionally with the TMJ. In particular, positional or volumetric changes occurring in the teeth or masticatory muscles may alter TMJ biomechanics [6]. In addition, it has been reported that occlusal forces generated during mastication can induce condylar growth through mechanical compression, leading to alterations in condylar morphology [7,8]. Abnormalities in muscle tone or disturbances in occlusal relationships have also been shown to produce uneven force distribution across the joint surfaces, thereby triggering adaptive remodeling processes [8,9,10]. Therefore, any disturbance affecting the stomatognathic system has the potential to induce changes in TMJ morphology and function [6].

The masticatory system and the distribution of occlusal forces may be significantly altered in conditions such as missing teeth, malocclusions, or impacted teeth. In this context, the role of the canine teeth in functional occlusion is critical. The principle of canine-protected occlusion, widely accepted in gnathology, states that during lateral mandibular movements, contact should occur exclusively on the canine teeth. This concept is regarded as the ideal functional occlusion model for individuals with natural dentition and is recommended as an important criterion for planning orthodontic treatment [11,12]. Impaction of the canine teeth may disrupt canine-protected occlusion and lead to deficiencies within the dental arch. Consequently, occlusal force transmission may be redirected toward the posterior teeth and indirectly toward the TMJ. This altered force distribution may increase condylar loading and, over time, induce morphological changes in the TMJ. Indeed, previous studies have reported that individuals lacking canine-protected occlusion exhibit an increased susceptibility to temporomandibular disorders (TMD) and post-treatment orthodontic relapse [13,14]. Furthermore, Rodrigues et al. [15] demonstrated a significant association between condylar morphological changes and tooth loss, suggesting that alterations in dental arch integrity may influence temporomandibular joint adaptation. In this context, impacted canine teeth may be considered a form of functional tooth absence, as they do not participate in occlusal guidance or effective load distribution. This may lead to localized loss of occlusal support and altered masticatory force transmission, potentially affecting the biomechanical balance of the temporomandibular joint.

Therefore, advanced imaging techniques are required both to monitor potential morphological alterations in the TMJ and to accurately determine the position of impacted canines. Two-dimensional radiographic techniques are limited in evaluating three-dimensional anatomical structures due to distortion, superimposition, and artifacts [16]. These limitations reduce measurement accuracy and compromise data validity, particularly for complex structures such as the TMJ and impacted teeth. In this regard, cone beam computed tomography (CBCT), which is widely used in orthodontics, enables detailed three-dimensional assessment of the craniofacial complex while offering advantages such as reduced radiation dose and cost-effectiveness [17,18]. The use of axial, coronal, and sagittal CBCT sections enables precise evaluation of both TMJ morphology and the intraosseous position of impacted canines. Consequently, the present study was conducted using CBCT data.

Despite the increasing use of CBCT for the evaluation of both impacted canines and TMJ morphology, the relationship between impacted canines and TMJ morphology remains insufficiently clarified. In particular, it is still unclear whether maxillary or mandibular canine impaction differentially affects TMJ morphology, as only a limited number of studies have directly investigated this association. Moreover, to the best of our knowledge, no previous study has simultaneously evaluated the effects of both maxillary and mandibular impacted canines on TMJ morphology within a single analytical framework. Therefore, the aim of this study was to evaluate TMJ morphology in individuals with impacted maxillary or mandibular canines using CBCT and to compare the findings with those of a control group without impacted canines. Accordingly, the following null hypotheses were tested:

**H01.**  *TMJ* *morphology does not differ significantly between individuals with impacted maxillary or mandibular canines and individuals without impacted canines.*

**H02.**  *In individuals with unilateral impacted canines, no significant difference exists between the impacted and non-impacted sides with respect to condylar morphological measurements.*

## 2. Materials and Methods

This retrospective study was conducted using CBCT images of individuals who applied to the Department of Orthodontics, Faculty of Dentistry, İnönü University, and were identified as having impacted canines in the archival records. The study protocol was approved by the Scientific Research Ethics Committee of İnönü University (Approval No: 2025/8171 and date of approval 29 July 2025).

### 2.1. Inclusion Criteria

Individuals meeting the following criteria were included in the study:

• Individuals with a unilateral impacted mandibular canine;
• Individuals with a unilateral impacted maxillary canine;
• Individuals without impacted canines in either the maxillary or mandibular regions (control group);
• Absence of impaction in teeth other than third molars;
• CBCT images of sufficient diagnostic quality;
• No history of previous orthodontic treatment;
• Absence of missing or supernumerary teeth;
• Absence of any structural anomaly or syndrome affecting the head and neck region;
• Age of 12 years or older.

### 2.2. Exclusion Criteria

Individuals meeting the following criteria were excluded from the study:

• Presence of impacted canines in both the maxillary and mandibular regions (to minimize confounding effects);
• CBCT records with insufficient diagnostic quality or the presence of imaging artifacts;
• History of orthodontic treatment;
• Age younger than 12 years;
• Presence of missing or supernumerary teeth;
• History of syndrome, trauma, or pathology affecting the temporomandibular joint or mandible.

Based on the position of the impacted canine detected on CBCT images, the participants were divided into three groups:

• Group 1: Individuals with an impacted maxillary canine;
• Group 2: Individuals with an impacted mandibular canine;
• Group 3 (Control): Individuals without impacted canines.

### 2.3. TMJ Measurements

**Figure 1:**
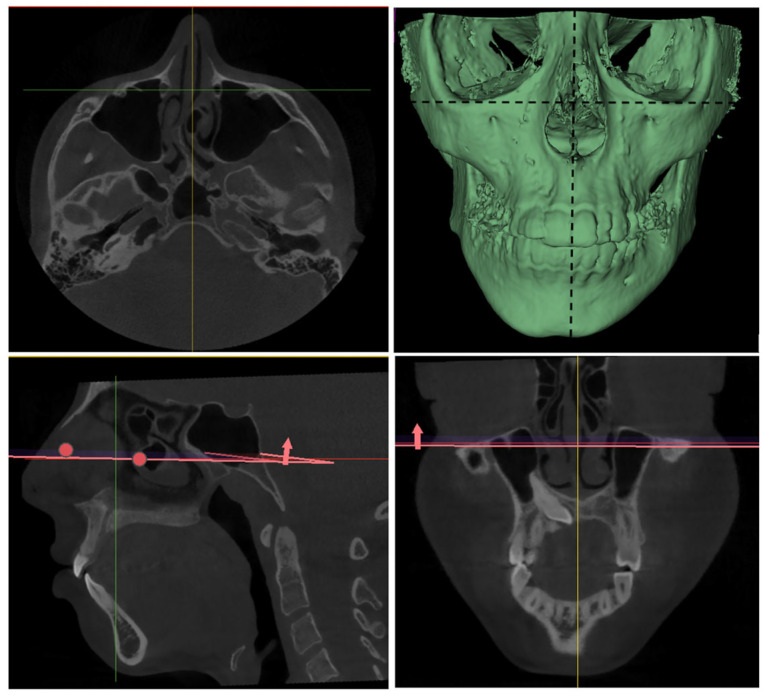
CBCT images were oriented with the Frankfurt Horizontal (FH) plane parallel to the ground and properly aligned with the midsagittal plane.

Following the determination of the sample size, the methodological workflow of the study was initiated, with a focus on the processing of CBCT data and the execution of morphometric measurements. In this context, CBCT images of the identified patients retrieved from the archival records were obtained in Digital Imaging and Communications in Medicine (DICOM) format and prepared for analysis. As a preliminary step in the analysis process, the DICOM datasets were imported into the 3D Slicer software (version 5.8.1). Prior to analysis, the CBCT images were standardised to a common reference position during the import process into 3D Slicer. Image orientation was first adjusted according to the Frankfurt Horizontal (FH) plane, followed by alignment to the midsagittal plane in the frontal view to ensure midline symmetry (Figure 1).

**Figure 2:**
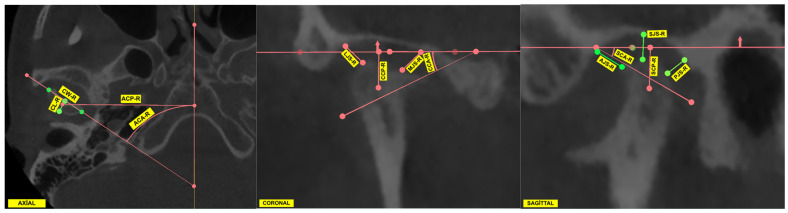
Axial, coronal, and sagittal cone beam computed tomography (CBCT) images illustrating the measurement protocol for condylar morphology and joint spaces. Axial view: Condylar width (CW), condylar length (CL), axial condylar position (ACP), and axial condylar angle (ACA). Coronal view: Lateral and medial joint spaces (LJS, MJS), coronal condylar position (CCP), and coronal condylar angle (CCA). Sagittal view: Superior, anterior, and posterior joint spaces (SJS, AJS, PJS), sagittal condylar position (SCP), and sagittal condylar angle (SCA). All measurements were performed relative to standardized reference planes, including the Frankfurt Horizontal (FH) and midsagittal planes.

Following completion of the orientation process, measurements were performed in the axial, sagittal, and coronal planes. In the context of temporomandibular joint (TMJ) assessment, linear morphometric parameters were the focus of the evaluation. These parameters included condylar width, condylar length, condylar position, and joint space measurements. In addition, condylar angular measurements were obtained in the axial, coronal, and sagittal planes. All measurements were calculated separately for the right and left condyles in each individual. The complete set of morphometric parameters used in the study is summarized in Table 1. All measurements were conducted in 3D Slicer, utilising predefined anatomical reference landmarks in accordance with a standardised measurement protocol (Figure 2).

### 2.4. TMJ Volume Measurements

**Figure 3:**
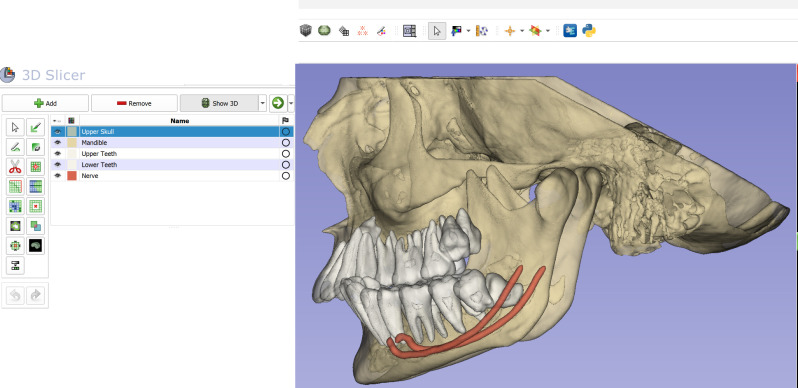
Segmentation of craniofacial structures from DICOM files using the DentalSegmentator AI module in 3D Slicer software (version 5.7).

After completion of the linear and angular measurements, condylar volume assessments were performed. For this purpose, the DICOM datasets of each patient were segmented into the mandible, maxilla and upper cranial structures, maxillary teeth, mandibular teeth, and the mandibular canal using the DentalSegmentator tool, an artificial intelligence (AI)-based extension developed by Dot et al. [19] (Figure 3).

**Figure 4:**
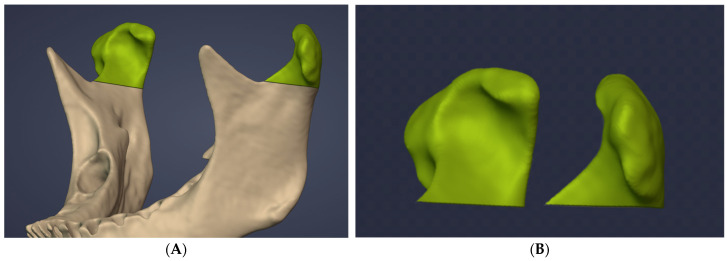
Separation of the mandibular condyle using the CUT tool in Blender for Dental: (**A**) condyle segmented from the mandible, (**B**) isolated three-dimensional condylar models.

Following the segmentation of the anatomical structures, the resulting STL files were transferred to Blenderfordental (B4D) software (Blenderfordental 2024, Dubai, United Arab Emirates) using the Blender Slicer Bridge Tool, which facilitates data exchange between 3D Slicer and Blenderfordental. Subsequent condylar volume measurements were performed in the B4D environment. In accordance with previous studies, the CUT tool within the Guide Module of the B4D software was used to define the inferior boundary of the condyle at the level where the sigmoid notch disappears, thereby isolating the condyle for volumetric analysis (Figure 4) [20,21].

Following the conclusion of all measurements, the intra-observer reliability was assessed by the repetition of the measurements in 10 randomly selected individuals by the same examiner (F.O.) at a two-week interval. Inter-observer reliability was evaluated by a second examiner (S.Ö.), who independently performed the measurements in 10 randomly selected individuals from each group using the same methodology. All obtained data were recorded in an Excel spreadsheet (Microsoft Office 365, Redmond, Washington, DC, USA).

### 2.5. Statistical Analysis

An a priori power analysis was conducted to determine the minimum required sample size for comparisons among the three study groups using G*Power software (version 3.1; Heinrich Heine University of Düsseldorf, Düsseldorf, Germany). The significance level (α) was set at 0.05, statistical power (1 − β) at 0.80, and effect size (Cohen’s f) at 0.40 [22]. Based on these parameters, the minimum required total sample size was calculated as 66 individuals. The sample size of 80 participants included in the present study was therefore considered sufficient to detect potential differences among the groups with adequate statistical power. A post hoc power analysis based on the primary outcome variable (condylar volume) was also conducted to verify that the final sample size provided adequate statistical power. Descriptive characteristics of the study sample are presented in Table 2.

Descriptive statistics, including the mean, standard deviation, median, minimum, maximum, Q1 and Q3, were calculated for all variables. The normality of data distribution was assessed using the Shapiro–Wilk test, and homogeneity of variances was evaluated using Levene’s test. For variables meeting the assumptions of normality and homogeneity of variance, one-way ANOVA was performed, followed by Tukey’s HSD test for post hoc pairwise comparisons. In variables where these assumptions were not met, the Kruskal–Wallis test was employed, with Dunn’s test subsequently applied with Bonferroni correction for post hoc multiple comparisons. Paired comparisons between right and left sides were conducted using paired *t*-test or Wilcoxon signed-rank test, depending on the normality of the differences. Pearson correlation coefficients were calculated in order to evaluate the relationships among TMJ parameters. Intra-observer and inter-observer reliability were assessed using intraclass correlation coefficients (ICCs, two-way random, absolute agreement). Statistical significance was set at *p* < 0.05. All statistical analyses and graphical visualizations were performed using RStudio (version 2025.09.2; Posit Software, PBC, Boston, MA, USA).

## 3. Results

A total of 80 subjects were evaluated, including 30 patients with maxillary impacted canines, 20 patients with mandibular impacted canines, and 30 control subjects. Descriptive measurements, normality tests, omnibus comparisons, pairwise analyses, and reliability estimates are summarized below.

**Figure 5:**
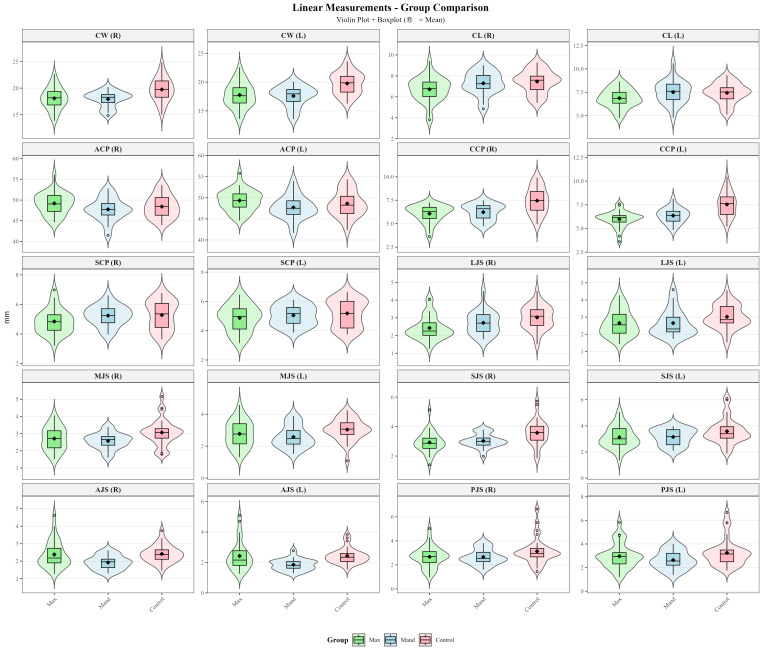
Violin plots of linear TMJ measurements across the study groups.

**Figure 6:**
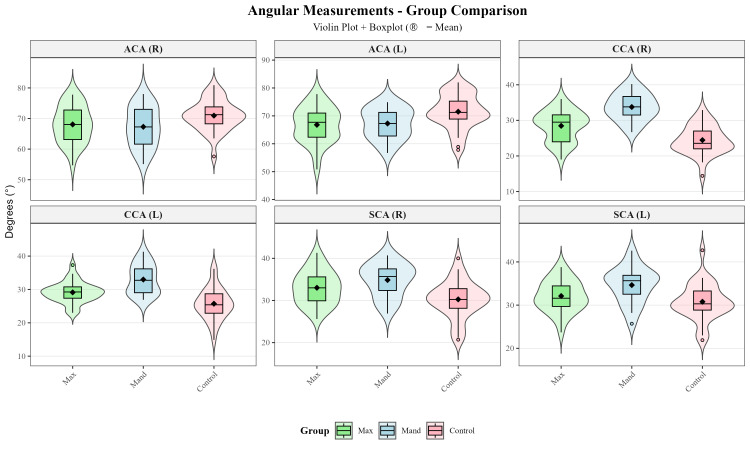
Violin plots of angular TMJ measurements across the study groups.

**Figure 7:**
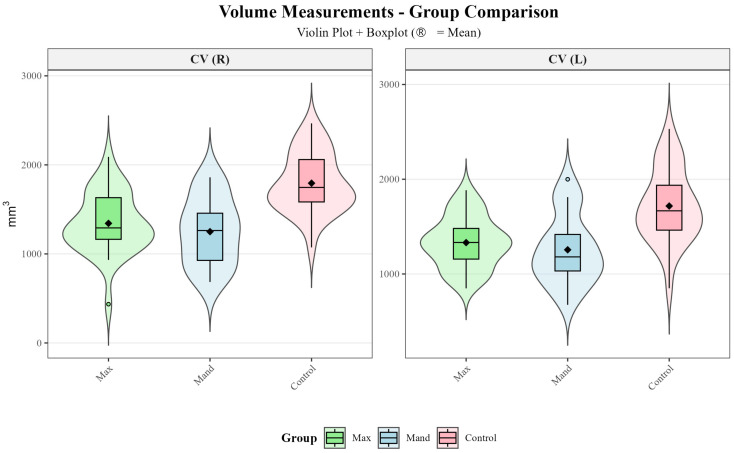
Violin plots of volumetric TMJ measurements across the study groups.

Violin plots combined with boxplots were used to visualize the distribution and central tendency of linear, angular and volumetric TMJ measurements across the maxillary impacted canine, mandibular impacted canine, and control groups (Figure 5, Figure 6 and Figure 7).

**Figure 8:**
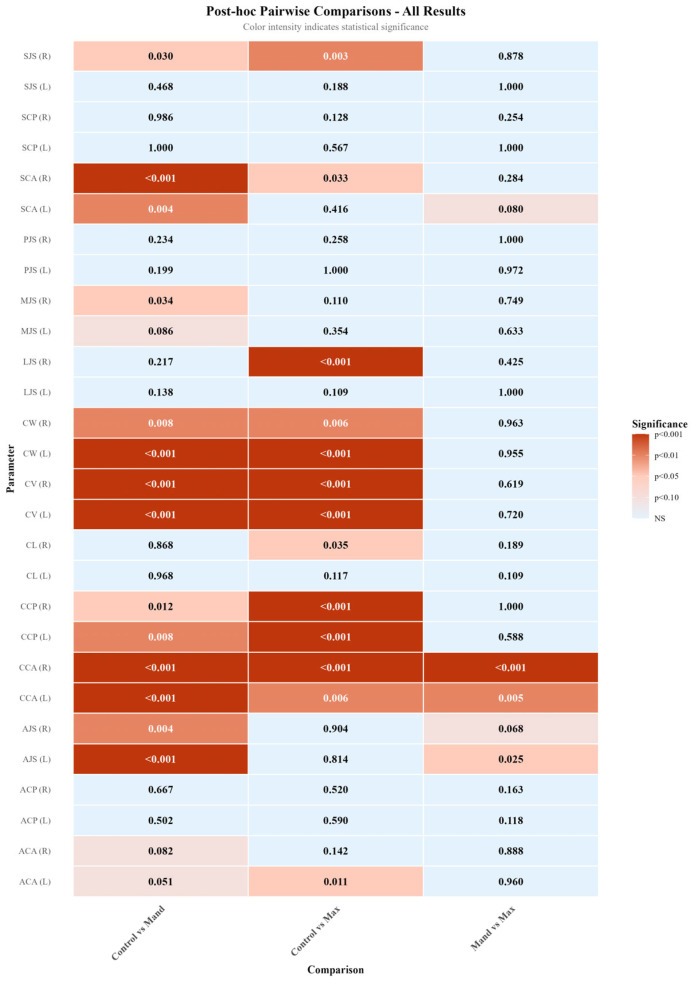
Comprehensive heatmap of adjusted *p*-values from post hoc pairwise comparisons, illustrating both significant and non-significant group differences.

Three-group comparisons using either one-way ANOVA or Kruskal–Wallis test (Table 3) revealed statistically significant differences in 17 of the 28 TMJ parameters examined (CW-R, CW-L, CL-R, ACA-L, CCP-R, CCP-L, CCA-R, CCA-L, LJS-R, MJS-R, SCA-R, SCA-L, SJS-R, AJS-R, AJS-L, CV-R, CV-L). Post hoc pairwise comparisons (Table 4 and Figure 8) were performed using Tukey’s HSD test or Dunn’s test with Bonferroni correction to identify which groups differed significantly.

Paired comparisons between right and left sides (Table 5) showed statistically significant differences in 1 parameter (CV). The remaining parameters demonstrated no statistically significant lateral asymmetry (*p* > 0.05).

When all impacted canine patients were evaluated together (Table 6), a statistically significant difference between the impacted and non-impacted sides was observed only for the CCP parameter (*p* = 0.002). No statistically significant differences were found for any of the remaining measurements between the two sides (*p* > 0.05).

Intra-observer reliability ranged from good to excellent, with ICC values between 0.844 and 0.990. Inter-observer reliability was similarly high, ranging from good to excellent (ICC: 0.795–0.966). Overall, these results indicate high measurement consistency and excellent reproducibility of the TMJ morphometric assessments.

Pearson correlation analysis demonstrated several significant associations among temporomandibular joint morphological parameters. In particular, moderate to strong positive correlations were observed between condylar width and condylar length with condylar volume on both sides. In addition, low-to-moderate correlations were identified between certain joint space measurements and condylar positional and angular parameters. The largely similar correlation patterns observed for the right and left temporomandibular joints support the biological consistency of the measurements (Figure 8).

The results of the age-related correlation analysis demonstrated the presence of varying degrees of associations among TMJ morphological parameters (Supplementary Table S1 and Figure S1). Significant correlations were identified between age and condylar volume (CV) as well as condylar position measurements (CCP) (*p* < 0.05), while some linear measurements showed moderate associations with age. Conversely, angular and joint space measurements demonstrated an absence or insignificance of correlation with age. No statistically significant correlations were observed between age and the remaining parameters (*p* > 0.05).

In gender-based comparisons, males exhibited significantly higher values than females for CW (right and left), ACP (right and left), CCA (right and left), PJS (left), LJS (left), and CV (right) (*p* < 0.05) (Supplementary Table S2 and Figure S2). No statistically significant gender-related differences were observed for the remaining TMJ morphological parameters (*p* > 0.05).

To further account for the potential confounding effects of age and sex, an analysis of covariance (ANCOVA) was performed with age and sex included as covariates for all TMJ morphological parameters. After adjustment, significant group effects persisted for a limited number of variables, specifically the coronal condylar angle on both sides (CCA_R, CCA_L) and the anterior joint space measurements (AJS_R, AJS_L) (*p* < 0.05) (Supplementary Table S3). In contrast, most linear and joint space parameters no longer demonstrated significant group differences after covariate adjustment.

## 4. Discussion

The objective of this study was to evaluate the TMJ morphology of subjects with impacted maxillary and mandibular canines using CBCT, and to compare the findings with those of a control group without impacted teeth. Based on the results, the first null hypothesis, which proposed that the presence of an impacted canine has no effect on TMJ morphology, was rejected because significant differences were detected across several linear, angular, and volumetric parameters. Conversely, the second null hypothesis—that there was no difference between the right and left sides in unilateral impacted canine cases—was largely accepted, as no significant side-to-side asymmetry was identified, with the exception of condylar volume.

The literature indicates a close relationship between condylar morphology and the type of malocclusion [23,24]. In a study examining the association between skeletal malocclusion and condylar volume, condylar volume was reported to be significantly higher in skeletal Class III individuals than in Class I and Class II individuals [20]. Conversely, skeletal Class II individuals were reported to exhibit lower condylar volume values than Class I and Class III individuals [20]. However, other studies have reported no significant differences in condylar morphology or dimensions between skeletal Class II and Class III individuals [8,25]. Furthermore, the vertical growth pattern has been hypothesised to exert a crucial influence on condylar volume [21,26]. In this context, condylar volume has been reported to be significantly higher in mandibular hypodivergent individuals than in hyperdivergent and normodivergent individuals, whereas lower condylar volume values have been observed in hyperdivergent individuals [21,26]. When the prevalence of degenerative changes in the TMJ is considered, such structural differences appear to be more pronounced in specific skeletal patterns. In particular, radiographically identifiable degenerative joint changes have been documented to occur with greater frequency in individuals exhibiting skeletal and dental Class II malocclusion, in conjunction with retrognathic mandibule [10,27,28]. Collectively, these findings suggest that changes in condylar volume and morphology may be closely associated with the development of temporomandibular disorders. Therefore, these parameters should be considered during clinical evaluation, and all factors that may influence TMJ morphology and biomechanics should be examined in a comprehensive and careful manner.

The literature indicates that TMJ position and morphology are influenced by multiple factors, including gender, age, growth pattern, and intra-articular pathologies [10,15,29]. Therefore, the assessment of TMJ morphology is of critical importance for understanding the biomechanical balance of the joint and its capacity for functional adaptation. In this context, fundamental anatomical features such as condylar height, width, and length are emphasized as playing a key role in maintaining joint biomechanics and joint health [30,31]. Moreover, the use of standardized three-dimensional mandibular landmarks in CBCT imaging has been shown to provide highly reproducible assessments of condylar morphology, further supporting the reliability of landmark-based TMJ evaluations [32].

It has been reported that occlusal forces generated during mastication may lead to alterations in condylar morphology [7,8]. Because occlusal loading is closely linked to the functional equilibrium of the stomatognathic system, conditions such as tooth loss, malocclusion, and dental impaction may substantially modify force distribution and joint loading patterns. Impaction of canine teeth may disrupt canine-protected occlusion and result in deficiencies within the dental arch, which in turn may induce morphological changes in the TMJ. Indeed, Rodrigues et al. [15] reported a significant association between changes in condylar morphology and tooth loss. Consequently, impacted canines may lead to discontinuity of the dental arch, disruption of functional integrity, and subsequent alterations in biomechanical balance.

Maxillary canines represent the second most frequently impacted tooth group [1], whereas impaction of mandibular canines is relatively rare [4,5]. Impaction of maxillary or mandibular canines may compromise dental arch integrity, cause imbalances in occlusal relationships, and alter functional force transmission, thereby inducing changes in TMJ morphology. Investigation of such changes requires advanced imaging techniques. In the present study, CBCT was utilized because of its low radiation dose and cost advantages, as well as its ability to provide detailed three-dimensional evaluation of the craniofacial complex [17,18].

In the present study, intergroup comparisons revealed significant differences across multiple TMJ morphological parameters (Table 3). Among the parameters evaluated in the axial plane, condylar width (CW-R, *p* = 0.002; CW-L, *p* < 0.001), condylar length (CL-R, *p* = 0.036), and axial condylar angle (ACA-L, *p* = 0.008) showed significant differences, whereas axial condylar position (ACP, *p* > 0.05) did not. In the coronal plane, coronal condylar position and angle (CCP and CCA, *p* < 0.001), as well as lateral and medial joint spaces (LJS-R, *p* = 0.001; MJS-R, *p* = 0.028), demonstrated statistically significant differences. In the sagittal plane, sagittal condylar angle (SCA-R, *p* < 0.001; SCA-L, *p* = 0.006), superior joint space (SJS-R, *p* = 0.002), and anterior joint space (AJS-R, *p* = 0.005; AJS-L, *p* = 0.001) differed significantly between groups, whereas sagittal condylar position and posterior joint space (SCP and PJS, *p* > 0.05) showed no significant differences. Additionally, condylar volume (CV, *p* < 0.001) demonstrated a statistically significant difference among the groups.

Post hoc analyses revealed that these significant differences were largely driven by comparisons between the control group and the impacted canine groups, with the control group exhibiting higher values for the corresponding linear, angular, and volumetric parameters (Table 4 and Figure 8). In contrast, no statistically significant differences were observed between the maxillary and mandibular impacted canine groups for most parameters (*p* > 0.05). This finding suggests that alterations in TMJ morphology may be more strongly related to disruption of dental arch integrity and changes in occlusal force distribution associated with the presence of an impacted canine, rather than to its maxillary or mandibular location.

The literature indicates that occlusal forces can induce condylar growth through mechanical compression, thereby leading to morphological changes in the condyle [7,8]. Kurusu et al. [8] reported that individuals in the high occlusal force group exhibited condyles with larger and more rounded morphology in the lateral and posterior regions compared with those in the low occlusal force group. In individuals with low occlusal forces, reduced mechanical loading transmitted to the mandibular condylar surface during mastication resulted in pronounced morphological differences, particularly in the lateral and posterior directions. Inhibition of condylar growth in these regions led to marked dimensional reductions in condylar morphology [8]. In this context, the higher condylar volume and other morphometric parameters observed in the control group compared with the impacted canine groups may reflect adaptive condylar responses. These changes may be associated with reduced occlusal forces or altered force transmission resulting from the presence of impacted teeth.

Furthermore, alterations in condylar morphology have been demonstrated to be substantially associated with tooth loss [15,29]. Yalcin and Ararat [29] reported that flat-type condyles were observed at a higher frequency in partially edentulous individuals compared with totally edentulous individuals. In the same study, angular-type condyles were found to be more prevalent in both partial and total edentulism, whereas convex-type condyles were more commonly observed in fully dentate individuals [29]. These findings indicate that the disruption of dental arch integrity may have a significant impact on condylar morphology. Considering that impacted canines may functionally create deficiencies within the dental arch and alter occlusal force distribution, the significant morphological differences observed between the impacted canine groups and the control group in the present study are consistent with previous reports demonstrating an association between tooth loss and condylar morphology.

**Figure 9:**
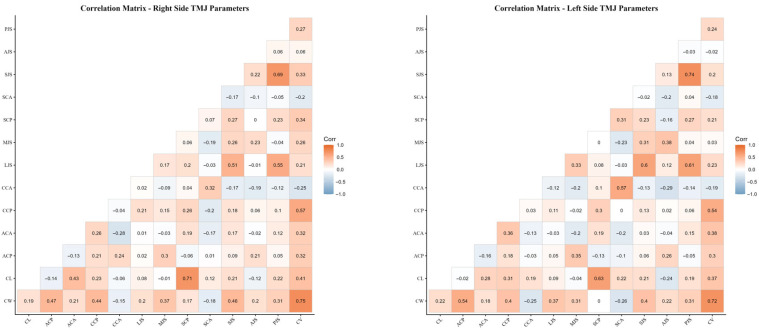
Correlation matrices of temporomandibular joint (TMJ) morphological parameters for the right and left sides.

In the present study, no statistically significant differences were detected between the right and left sides for the majority of TMJ morphological parameters (Table 5) (*p* > 0.05). In the groups with impacted canines, a statistically significant difference between the impacted and non-impacted sides was observed only for the CCP parameter (*p* = 0.002), whereas no significant side-to-side differences were found for the remaining parameters (*p* > 0.05) (Table 6). This finding suggests that, despite the presence of an impacted canine, the overall morphological structure of the TMJ largely remains bilaterally symmetric. Supporting this observation, correlation analysis revealed moderate to strong positive associations between morphometric parameters of the condyle—particularly condylar width and length—on both sides. Moreover, the largely similar correlation patterns observed for the right and left TMJs indicate that the dimensional parameters of condylar morphology change in a coordinated manner (Figure 9). Collectively, these findings suggest that the functional adaptation of the TMJ generally occurs through a bilateral and balanced morphometric response. On the other hand, the presence of a statistically significant difference in condylar volume between the right and left sides (*p* < 0.05) suggests that the adaptive response of the condyle to functional loading may manifest at the volumetric level. This observation indicates that condylar volume may represent a parameter that is more sensitive to functional changes compared with other linear and angular measurements.

Consistent with the findings of the present study, the literature generally reports a high degree of concordance and symmetry between right and left condylar morphological parameters [33,34,35]. It has been reported that condylar dimensions usually do not differ significantly between sides even in young adults with facial asymmetry [33]. Similarly, no side-to-side differences in condylar dimensions have been observed in children with posterior crossbite [34]. Marmary et al. [36] reported craniofacial structures exhibit significant midline symmetry and minor asymmetries may be considered within the range of normal asymmetry. However, the present study found condylar volume was found to be significantly greater on the left side compared with the right side (*p* < 0.05). In line with this finding, Cohlmia et al. [37] suggested that condylar position may be asymmetric even in a normal population. Similarly, Al-Koshab et al. [35] reported that condylar volume may vary between the right and left sides. Contrary to the conclusions of the present study, a previous investigation found that, while condylar volume exhibited variation in relation to disparate vertical growth patterns, a significant and positive correlation was identified between right and left condylar volumes [21]. This discrepancy may be related to the presence of different malocclusion types within the study sample. Additionally, the presence of a preferred chewing side in individuals with malocclusion has been proposed as a potential contributor to asymmetry in condylar volume [37].

When the relationships between age and TMJ morphological parameters were evaluated in the present study, strong and statistically significant correlations were identified particularly between age and condylar volume (CV) as well as condylar position measurements (CCP) (r = 0.507–0.576; *p* < 0.001). In addition, moderate correlations with age were observed for certain linear and angular parameters, including CW, CCA, and SCA (r ≈ 0.42–0.46; *p* < 0.001), whereas correlations involving joint space measurements were generally weak (r ≈ 0.22–0.32; *p* < 0.05). No statistically significant relationships were identified between age and the remaining parameters (*p* > 0.05) (Supplementary Table S1 and Figure S1).

The literature presents divergent findings regarding the relationship between age and mandibular condylar morphology. Rodrigues et al. [15] reported that changes in the mandibular condyle were significantly associated with age groups, whereas Assari et al. [38] reported no significant association between condylar height and age groups. In the present study, the effect of age on TMJ morphology appears to be parameter-specific. The significant associations observed between age and condylar volume as well as condylar position measurements suggest that the condyle may undergo morphological changes over time. Conversely, no substantial correlations were identified between age and angular or joint space measurements. These findings indicate that the influence of age on TMJ morphology is not uniform and may vary depending on the parameter evaluated.

In gender-based evaluations, males exhibited statistically significantly higher values than females for several linear, angular, and volumetric measurements, including CW, ACP, CCA, and CV (*p* < 0.05). However, no statistically significant gender-related differences were observed for the remaining measurements (*p* > 0.05) (Supplementary Table S2 and Figure S2). The literature reports inconsistent findings regarding the association between condylar morphology and gender. Some studies have indicated that condylar volume and dimensions are greater in males than in females, potentially related to post-pubertal differences in growth patterns and overall skeletal size [26,39,40]. In contrast, other studies have reported no significant association between condylar morphology and gender [15,41]. In the present study, the observation of higher values for certain condylar measurements in males is consistent with these heterogeneous findings and suggests that the influence of gender on condylar morphology may vary depending on the parameter evaluated and the characteristics of the study sample.

The present study has several limitations. First, due to its retrospective design, clinical TMJ symptoms, preferred chewing side, and functional habits could not be assessed, limiting the direct association between morphological findings and clinical outcomes. In addition, age- and sex-related differences among the study groups—together with the relatively higher mean age of the control group—may have influenced certain TMJ parameters, given the known effects of physiological remodeling and sexual dimorphism on condylar morphology. Furthermore, although impacted canines were considered the primary factor of interest, TMJ morphology may also be affected by other occlusal and skeletal characteristics, including sagittal and vertical skeletal patterns, occlusal interferences, overbite and overjet relationships, and overall malocclusion severity. Future prospective and longitudinal studies with age- and sex-matched groups, larger and more homogeneous samples, and stricter control of occlusal and skeletal variables are recommended to more clearly isolate the independent effects of impacted canines on TMJ morphology.

## 5. Conclusions

This study demonstrated that the presence of impacted canines may be associated with significant changes in certain linear, angular, and volumetric TMJ morphological parameters. While condylar width, condylar position and angulation, joint space measurements, and condylar volume were found to be higher in the control group compared with the impacted canine groups, no significant differences were observed for most parameters between the maxillary and mandibular impacted canine groups. These findings suggest that alterations in TMJ morphology may be more closely related to changes in occlusal force distribution associated with the presence of an impacted canine rather than to its maxillary or mandibular location.

In right–left comparisons, the preservation of bilateral symmetry in the majority of parameters, with the exception of condylar volume, in conjunction with analogous correlation patterns, lends support to the hypothesis that TMJ functional adaptation occurs in a bilateral and consistent manner. Furthermore, the significant associations observed between age and particularly condylar volume and condylar position measurements, as well as the higher condylar volume detected in males, indicate that age and sex may influence TMJ morphology.

In conclusion, evaluation of impacted canines should consider not only local dental effects but also the morphological characteristics of the temporomandibular joint, thereby enabling a more comprehensive approach to orthodontic diagnosis and treatment planning.

## Figures and Tables

**Table 1:** Condylar morphometric parameters and definitions.

Parameter (Abbreviation)	Description
Right–Left Condylar Width (CW-R/CW-L)	Mediolateral width of the condyle
Right–Left Condylar Length (CL-R/CL-L)	Anteroposterior length of the condyle
Right–Left Axial Condylar Position (ACP-R/ACP-L)	The mediolateral distance of the condyle from the midsagittal plane in the axial view
Right–Left Axial Condylar Angle (ACA-R/ACA-L)	The angle formed between the long axis of the condyle and the midsagittal plane in the axial view
Right–Left Coronal Condylar Position (CCP-R/CCP-L)	The vertical position of the condyle relative to the FH plane in the coronal view
Right–Left Coronal Condylar Angle (CCA-R/CCA-L)	The angle formed between the long axis of the condyle and the FH plane in the coronal view
Right–Left Lateral Joint Space (LJS-R)/LJS-L)	Distance between the lateral condylar surface and the articular fossa
Right–Left Medial Joint Space (MJS-R/MJS-L)	Distance between the medial condylar surface and the articular fossa
Right–Left Sagittal Condylar Position (SCP-R/SCP-L)	The spatial position of the condyle in the sagittal plane relative to the FH plane.
Right–Left Sagittal Condylar Angle (SCA-R/SCA-L)	The angle formed between the long axis of the condyle and the FH plane in the sagittal view.
Right–Left Superior Joint Space (SJS-R/SJS-L)	Distance between the condylar apex and the fossa roof
Right–Left Anterior Joint Space (AJS-R/AJS-L)	Distance between the anterior condylar surface and the anterior fossa wall
Right–Left Posterior Joint Space (PJS-R/PJS-L)	Distance between the posterior condylar surface and the posterior fossa wall
Right–Left Condylar Volume (CV-R/CV-L)	Volume of the condyle (mm^3^)

**Table 2:** Descriptive statistics for the groups.

Group	Number of Patients	Females	Males	Mean Age (Years ± SD)
Impact maxillary canine	30	15	15	16.8 ± 3.3
Impact mandibular canine	20	12	8	16.6 ± 5.0
Control Group	30	18	12	20.7 ± 3.0

SD: Standard Deviation.

**Table 3:** Summary of three-group comparisons for TMJ morphometric parameters.

Parameters	Test	*p*-Value (Right)	*p*-Value (Left)	Sig.
CW	ANOVA	0.002	<0.001	*
CL	ANOVA	0.036	0.065	*/ns
ACP	ANOVA	0.182	0.141	ns
ACA	ANOVA	0.060	0.008	ns/*
CCP	Kruskal–Wallis	<0.001	<0.001	*
CCA	ANOVA	<0.001	<0.001	*
LJS	Kruskal–Wallis	0.001	0.056	*/ns
MJS	ANOVA	0.028	0.095	*/ns
SCP	ANOVA/KW	0.112	0.418	ns
SCA	ANOVA	<0.001	0.006	*
SJS	ANOVA/KW	0.002	0.143	*/ns
AJS	Kruskal–Wallis	0.005	0.001	*
PJS	Kruskal–Wallis	0.124	0.183	ns
CV	ANOVA	<0.001	<0.001	*

* *p* < 0.05; ANOVA: One-way Analysis of Variance; KW: Kruskal–Wallis test.

**Table 4:** Summary of significant post hoc pairwise comparisons.

Parameter	Side	Significant Comparison	Adjusted *p*-Value	Post Hoc Test
CW	R/L	Control > Maxillary, Mandibular	0.006–<0.001	Tukey HSD
CL	R	Control > Maxillary	0.035	Tukey HSD
ACA	L	Control > Maxillary	0.011	Tukey HSD
CCP	R/L	Control > Maxillary, Mandibular	<0.001	Dunn (Bonferroni)
CCA	R/L	All pairwise comparisons	<0.001	Tukey HSD
LJS	R	Control > Maxillary	<0.001	Dunn (Bonferroni)
MJS	R	Control > Mandibular	0.034	Tukey HSD
SCA	R/L	Control > Mandibular	<0.001–0.004	Tukey HSD
SJS	R	Control > Maxillary, Mandibular	0.003–0.030	Tukey HSD
AJS	R/L	Control > Mandibular	0.004–<0.001	Dunn (Bonferroni)
CV	R/L	Control > Maxillary, Mandibular	<0.001	Tukey HSD

Only statistically significant post hoc comparisons are presented. *p* < 0.05 was considered statistically significant. Dunn tests were Bonferroni-corrected.

**Table 5:** Right–Left Side Comparisons of TMJ Morphological Parameters.

Variable	Right Side (Mean ± SD)	Left Side (Mean ± SD)	Test	*p*-Value
CW	18.66 ± 2.17	18.47 ± 2.22	Paired *t*-test	0.126
CL	7.14 ± 1.17	7.25 ± 1.12	Wilcoxon	0.411
ACP	48.54 ± 2.72	48.66 ± 2.87	Wilcoxon	0.575
ACA	68.95 ± 5.96	68.67 ± 6.47	Wilcoxon	0.421
CCP	6.63 ± 1.27	6.66 ± 1.24	Wilcoxon	0.396
CCA	28.28 ± 5.41	28.81 ± 4.98	Wilcoxon	0.309
LJS	2.72 ± 0.71	2.79 ± 0.75	Wilcoxon	0.214
MJS	2.81 ± 0.70	2.83 ± 0.75	Wilcoxon	0.866
SCP	5.11 ± 0.88	5.03 ± 0.88	Wilcoxon	0.096
SCA	32.45 ± 4.51	32.25 ± 4.25	Paired *t*-test	0.646
SJS	3.20 ± 0.78	3.30 ± 0.86	Wilcoxon	0.112
AJS	2.28 ± 0.62	2.28 ± 0.74	Wilcoxon	0.606
PJS	2.84 ± 0.94	2.99 ± 1.06	Wilcoxon	0.065
CV	1457.09 ± 393.15	1489.05 ± 416.45	Wilcoxon	0.016 *

Paired *t*-test or Wilcoxon signed-rank test was used depending on normality. * Statistically significant at *p* < 0.05.

**Table 6:** Impacted–Non-Impacted Side Comparisons of TMJ Morphological Parameters.

Variable	Impacted (Mean ± SD)	Non-Impacted (Mean ± SD)	Test	*p*-Value
CW	17.80 ± 1.90	17.91 ± 2.00	Wilcoxon	0.423
CL	7.04 ± 1.12	7.04 ± 1.23	Paired *t*-test	0.992
ACP	48.76 ± 2.82	48.53 ± 2.66	Paired *t*-test	0.145
ACA	67.07 ± 6.76	67.67 ± 5.44	Paired *t*-test	0.388
CCP	6.00 ± 0.93	6.26 ± 0.80	Paired *t*-test	0.002 *
CCA	30.57 ± 4.66	30.68 ± 4.32	Paired *t*-test	0.865
LJS	2.69 ± 0.69	2.50 ± 0.73	Wilcoxon	0.167
MJS	2.72 ± 0.67	2.64 ± 0.72	Paired *t*-test	0.423
SCP	5.00 ± 0.87	4.95 ± 0.78	Paired *t*-test	0.663
SCA	33.48 ± 4.31	33.40 ± 3.79	Paired *t*-test	0.904
SJS	3.09 ± 0.65	3.01 ± 0.74	Wilcoxon	0.992
AJS	2.25 ± 0.84	2.14 ± 0.67	Wilcoxon	0.325
PJS	2.79 ± 0.79	2.71 ± 0.97	Wilcoxon	0.858
CV	1296.63 ± 350.85	1310.15 ± 309.76	Wilcoxon	0.946

Paired *t*-test or Wilcoxon signed-rank test was used depending on normality. * Statistically significant at *p* < 0.05.

## Data Availability

The original contributions presented in the study are included in the article; further inquiries can be directed to the corresponding author.

## Supplementary Materials

The following supporting information can be downloaded at: https://www.mdpi.com/article/10.3390/diagnostics16030496/s1, Table S1. Age-Parameter Correlations. Table S2. Gender Comparison of TMJ Parameters. Table S3. Analysis of Covariance (ANCOVA) Results Adjusted for Age and Sex. Figure S1. Heatmap illustrating the correlations between age and TMJ morphological parameters. Figure S2. Heatmap of gender-based comparisons of TMJ parameters showing *p*-value distributions and direction of differences.

## Abbreviations

TMJ	Temporomandibular Joint
CBCT	Cone Beam Computed Tomography
FH	Frankfort Horizontal
DICOM	Digital Imaging and Communications in Medicine
ICC	Intraclass Correlation Coefficient
AI	Artificial Intelligence
