# Supplementary material for: Three-Dimensional Evaluation of TMJ Morphology in Individuals with Maxillary or Mandibular Impacted Canines: A CBCT-Based Retrospective Study
Source: Diagnostics (Basel). 2026 Feb 6;16(3):496. doi: 10.3390/diagnostics16030496 (PMC12896977; doi:10.3390/diagnostics16030496)
Supplement: Supplementary file 1 [file diagnostics-16-00496-s001.zip › diagnostics-4084925-supplementary.pdf]

Supplementary Table S1. Age-Parameter Correlations

Parameter	Correlation (r)	p-value	Method	Strength	Sig.
CV (R)	0.576	<0.001	Pearson	Strong	*
CCP (L)	0.539	<0.001	Spearman	Strong	*
CV (L)	0.535	<0.001	Pearson	Strong	*
CCP (R)	0.507	<0.001	Pearson	Strong	*
CCA (R)	-0.503	<0.001	Pearson	Strong	*
CW (L)	0.463	<0.001	Pearson	Moderate	*
CCA (L)	-0.444	<0.001	Pearson	Moderate	*
CW (R)	0.432	<0.001	Pearson	Moderate	*
SCA (R)	-0.425	<0.001	Pearson	Moderate	*
LJS (R)	0.316	0.004	Pearson	Moderate	*
SCA (L)	-0.313	0.005	Pearson	Moderate	*
SJS (R)	0.309	0.005	Spearman	Moderate	*
ACA (R)	0.294	0.008	Pearson	Weak	*
MJS (R)	0.290	0.009	Pearson	Weak	*
ACA (L)	0.283	0.011	Pearson	Weak	*
AJS (R)	0.237	0.034	Spearman	Weak	*
LJS (L)	0.223	0.047	Spearman	Weak	*
MJS (L)	0.191	0.089	Pearson	Weak	ns
AJS (L)	0.190	0.092	Spearman	Weak	ns
CL (L)	0.183	0.104	Pearson	Weak	ns
CL (R)	0.181	0.109	Pearson	Weak	ns
SJS (L)	0.135	0.231	Spearman	Weak	ns
SCP (R)	0.134	0.235	Pearson	Weak	ns
PJS (R)	0.096	0.399	Spearman	Weak	ns
SCP (L)	0.058	0.609	Spearman	Weak	ns
ACP (L)	0.058	0.610	Pearson	Weak	ns
PJS (L)	0.047	0.682	Spearman	Weak	ns
ACP (R)	0.016	0.886	Pearson	Weak	ns

Ns: not statistically significant ($p > 0.05$). * indicates $p < 0.05$. Correlation strength: Weak (<0.3), Moderate (0.3-0.5), Strong (0.5-0.7), Very Strong (>0.7)

Supplementary Table S2. Gender Comparison of TMJ Parameters

Parameter	Male (Mean ± SD)	Female (Mean ± SD)	p-value	Test	Sig.
CW (R)	19.47 ± 2.04	18.04 ± 2.08	0.003	t-test	*
ACP (R)	49.49 ± 2.10	47.80 ± 2.93	0.004	t-test	*
ACP (L)	49.65 ± 2.30	47.88 ± 3.05	0.004	t-test	*
CCA (L)	30.54 ± 4.87	27.47 ± 4.69	0.006	t-test	*
CW (L)	19.17 ± 2.03	17.93 ± 2.24	0.012	t-test	*
CCA (R)	29.79 ± 4.63	27.11 ± 5.73	0.024	t-test	*
PJS (L)	3.29 ± 1.23	2.76 ± 0.85	0.028	Mann-Whitney	*
LJS (L)	2.96 ± 0.75	2.66 ± 0.73	0.030	Mann-Whitney	*
CV (R)	1594.73 ± 400.54	1406.85 ± 414.26	0.044	t-test	*
PJS (R)	3.10 ± 1.08	2.64 ± 0.77	0.066	Mann-Whitney	ns
SJS (L)	3.52 ± 0.89	3.13 ± 0.81	0.073	Mann-Whitney	ns
CL (L)	7.48 ± 1.08	7.07 ± 1.14	0.101	t-test	ns
CV (L)	1529.27 ± 384.28	1400.95 ± 395.02	0.148	t-test	ns
SJS (R)	3.38 ± 0.87	3.05 ± 0.69	0.150	Mann-Whitney	ns
LJS (R)	2.84 ± 0.65	2.63 ± 0.74	0.180	t-test	ns
CCP (L)	6.74 ± 1.20	6.59 ± 1.28	0.206	Mann-Whitney	ns
CL (R)	7.31 ± 1.10	7.01 ± 1.21	0.259	t-test	ns
MJS (L)	2.93 ± 0.83	2.74 ± 0.69	0.274	t-test	ns
AJS (R)	2.21 ± 0.57	2.33 ± 0.67	0.311	Mann-Whitney	ns
CCP (R)	6.76 ± 1.21	6.53 ± 1.33	0.404	Mann-Whitney	ns
SCA (R)	32.89 ± 4.45	32.10 ± 4.58	0.443	t-test	ns
ACA (R)	69.51 ± 5.27	68.52 ± 6.47	0.452	t-test	ns
MJS (R)	2.87 ± 0.77	2.77 ± 0.64	0.538	t-test	ns
SCP (R)	5.17 ± 0.80	5.06 ± 0.94	0.591	t-test	ns
SCP (L)	5.00 ± 0.87	5.06 ± 0.90	0.776	t-test	ns
SCA (L)	32.36 ± 4.12	32.16 ± 4.39	0.837	t-test	ns
AJS (L)	2.29 ± 0.72	2.28 ± 0.76	0.869	Mann-Whitney	ns
ACA (L)	68.62 ± 6.61	68.71 ± 6.44	0.951	t-test	ns

Ns: not statistically significant ($p > 0.05$). * indicates $p < 0.05$. SD: Standard Deviation.

Supplementary Table S3. Analysis of Covariance (ANCOVA) Results Adjusted for Age and Sex.

Variable	Adj. Mean Max (±SD)	Adj. Mean Mand(±SD)	Adj. Mean Control (±SD)	F (Group)	p (Group)	F (Age)	p (Age)	F (Sex)	p (Sex)	Sig.
CW_R	18.61 ± 0.42	18.64 ± 0.49	18.99 ± 0.54	0.108	0.898	4.543	0.036	13.839	<0.001	
CW_L	18.15 ± 0.43	18.14 ± 0.50	19.24 ± 0.55	0.900	0.411	2.423	0.124	10.392	0.002	
CL_R	6.73 ± 0.26	7.34 ± 0.30	7.48 ± 0.33	2.316	0.106	0.004	0.949	2.002	0.161	
CL_L	7.06 ± 0.24	7.76 ± 0.29	7.17 ± 0.32	2.615	0.080	1.650	0.203	3.894	0.052	
ACP_R	49.46 ± 0.59	48.20 ± 0.69	48.12 ± 0.76	1.699	0.190	0.612	0.437	7.512	0.008	
ACP_L	49.77 ± 0.62	48.32 ± 0.72	48.06 ± 0.80	2.188	0.119	1.371	0.245	7.571	0.007	
ACA_R	69.06 ± 1.32	68.45 ± 1.54	69.36 ± 1.71	0.088	0.916	1.612	0.208	0.781	0.380	
ACA_L	66.71 ± 1.42	67.21 ± 1.66	71.64 ± 1.84	1.572	0.214	0.007	0.931	0.019	0.890	
CCP_R	6.31 ± 0.25	6.51 ± 0.29	7.09 ± 0.32	1.305	0.277	2.507	0.118	1.766	0.188	
CCP_L	6.18 ± 0.23	6.59 ± 0.27	7.22 ± 0.30	2.995	0.056	2.100	0.152	1.334	0.252	
CCA_R	28.10 ± 0.88	33.65 ± 1.03	25.33 ± 1.14	16.302	<0.001	0.474	0.493	9.221	0.003	*
CCA_L	28.82 ± 0.88	32.95 ± 1.03	26.56 ± 1.14	9.245	<0.001	0.358	0.551	11.949	<0.001	*
LJS_R	2.49 ± 0.15	2.81 ± 0.18	2.94 ± 0.19	2.112	0.128	0.498	0.482	3.037	0.085	
LJS_L	2.71 ± 0.16	2.74 ± 0.19	2.96 ± 0.21	0.292	0.747	0.334	0.565	4.068	0.047	
MJS_R	2.79 ± 0.15	2.66 ± 0.18	2.96 ± 0.20	0.474	0.624	0.637	0.427	0.590	0.445	
MJS_L	2.78 ± 0.17	2.61 ± 0.20	3.05 ± 0.22	0.867	0.424	0.003	0.955	1.432	0.235	
SCP_R	4.86 ± 0.20	5.27 ± 0.23	5.28 ± 0.26	1.586	0.212	0.011	0.918	0.553	0.460	
SCP_L	4.81 ± 0.20	4.99 ± 0.24	5.28 ± 0.26	0.768	0.468	0.214	0.645	0.027	0.869	
SCA_R	31.96 ± 0.93	33.81 ± 1.09	32.13 ± 1.21	1.247	0.293	3.626	0.061	0.528	0.470	
SCA_L	31.31 ± 0.91	33.83 ± 1.07	32.16 ± 1.18	2.339	0.103	2.161	0.146	0.049	0.826	
SJS_R	2.93 ± 0.16	3.07 ± 0.19	3.62 ± 0.21	2.329	0.104	0.000	0.999	5.343	0.024	
SJS_L	3.11 ± 0.19	3.18 ± 0.22	3.64 ± 0.24	1.009	0.370	0.007	0.932	4.989	0.028	
AJS_R	2.44 ± 0.14	1.97 ± 0.16	2.30 ± 0.18	3.650	0.031	0.566	0.454	1.031	0.313	*
AJS_L	2.46 ± 0.16	1.89 ± 0.19	2.37 ± 0.21	4.018	0.022	0.148	0.702	0.012	0.913	*
PJS_R	2.58 ± 0.20	2.61 ± 0.24	3.33 ± 0.26	1.835	0.167	0.563	0.455	5.821	0.018	
PJS_L	2.81 ± 0.23	2.53 ± 0.27	3.56 ± 0.30	2.325	0.105	1.228	0.271	5.404	0.023	
CV_R	1430.19 ± 73.36	1363.23 ± 85.98	1667.50 ± 95.00	2.003	0.142	3.990	0.049	8.575	0.005	
CV_L	1418.92 ± 74.31	1362.74 ± 87.09	1583.34 ± 96.23	1.026	0.363	3.939	0.051	4.154	0.045	

ANCOVA: Analysis of Covariance with Age and Sex as covariates. Adj. Mean: Adjusted (estimated marginal) means. * $p < 0.05$. SD: Standard Deviation.

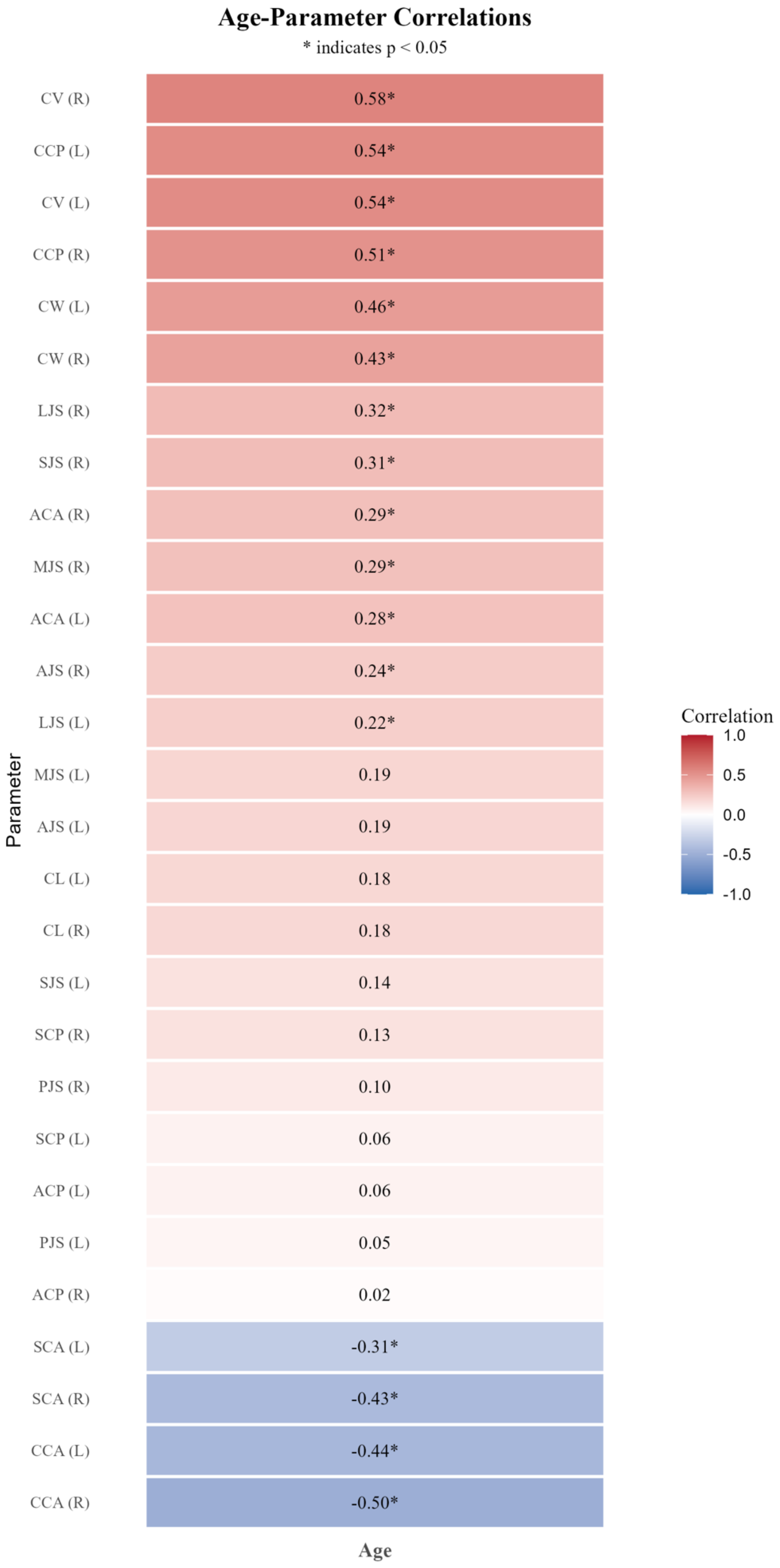

Supplementary Figure S1. Heatmap illustrating the correlations between age and TMJ morphological parameters. Positive correlations are shown in red and negative correlations in blue. * statistically significant correlations ($p < 0.05$).

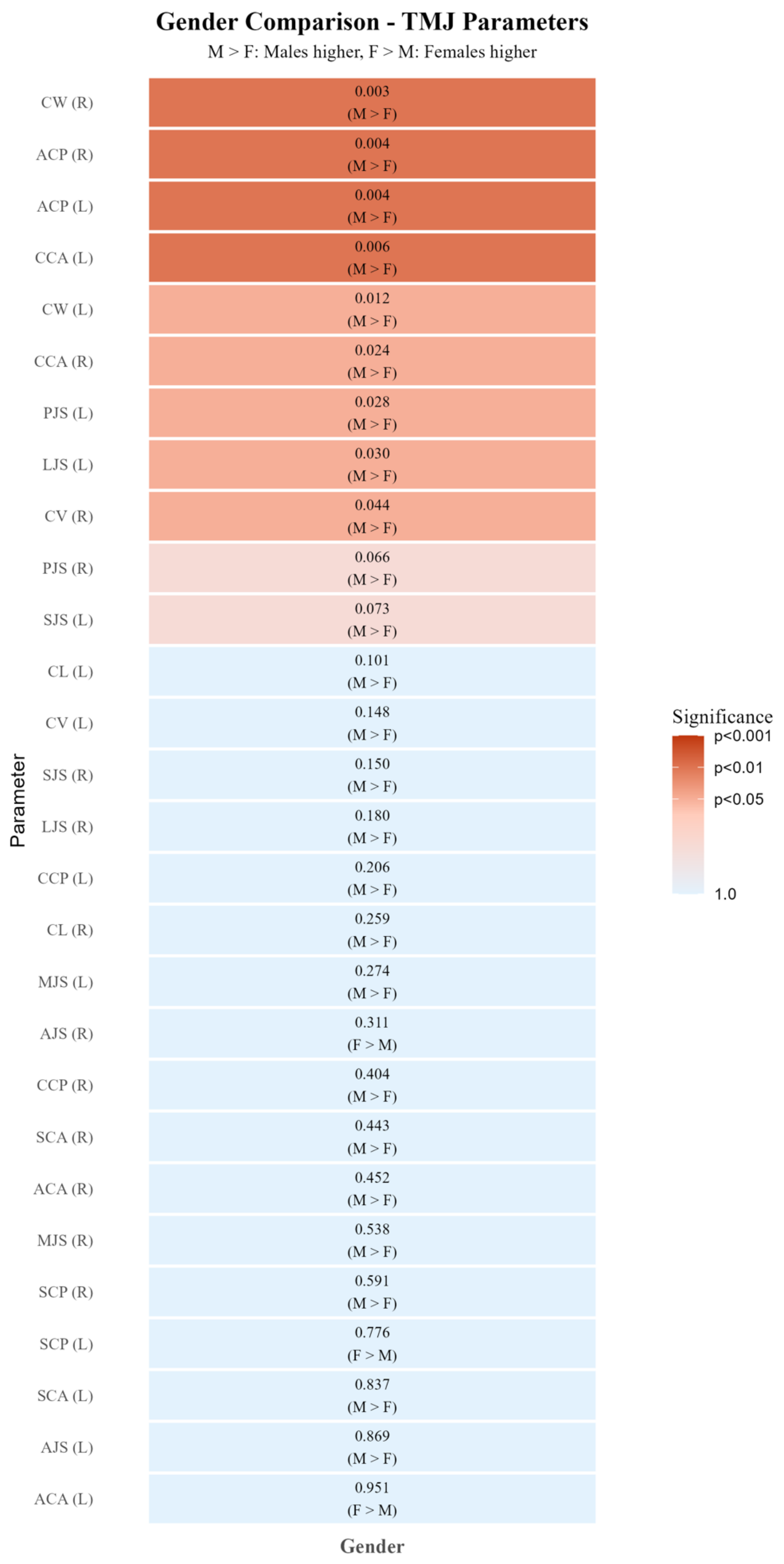

Supplementary Figure S2. Heatmap of gender-based comparisons of TMJ parameters showing p-value distributions and direction of differences.
